# Medial pelvic migration of the lag screw in a short gamma nail after hip fracture fixation: a case report and review of the literature

**DOI:** 10.1186/1749-799X-5-62

**Published:** 2010-08-27

**Authors:** Xinning Li, Michael J Heffernan, Christina Kane, Walter Leclair

**Affiliations:** 1 University of Massachusetts Medical Center, Worcester, MA, USA

## Abstract

Hip fractures are a common injury among the elderly. Internal fixation with an intramedullary (IM) system has gained popularity for the treatment of intertrochanteric femur fractures. Multiple complications associated with IM fracture fixation have been described, however, we report a rare complication of medial pelvic migration of the lag screw of a short IM nail in a stable construct ten weeks post surgery. The patient was subsequently treated with Lag Screw removal and revision surgery with a shorter Lag Screw and an accessory cannulated screw acting as a de-rotational device. The patient did well with the revision surgery and was able to return to full activities.

## Background

Hip fractures are a common injury among adults ages 65 and older, occurring in approximately 300,000 individuals yearly [1,2]. An estimated 1.66 million hip fractures occurred worldwide in 1990 and the number is expected to increase to 6.26 million by the year 2050 [3]. Internal fixation with an intramedullary (IM) system has gained popularity for the treatment of intertrochanteric femur fractures. This construct provides both load sharing properties while allowing immediate mobilization [4,5]. Complications of IM hip fracture fixation include but not limited to infection, mal or non union, avascular necrosis, hardware failure, neurovascular injuries, fracture of the femur distal to the implant, and death [3-5].

A common complication associated with the IM system is lag screw cut-out from the femoral head which leads to varus collapse of the fracture and non-union [6-8]. Other rare complications related to the lag screw include medial pelvic migration [9], lateral migration [10], extracorporeal extrusion from the body [11], and sigmoid perforation [8]. Furthermore, medial migration of the proximal femoral element seen with the Proximal Femoral Nail described as the "Z Effect" have also been reported [12]. We believe this is the first case report presenting medial pelvic migration of the lag screw or femoral neck element of a short Gamma 3 IM nail in a locked and stable construct ten weeks post surgery. The patient was subsequently treated with Lag Screw removal and revision surgery with a shorter Lag Screw and an accessory cannulated screw acting as a de-rotational device.

## Case presentation

**Figure 1:**
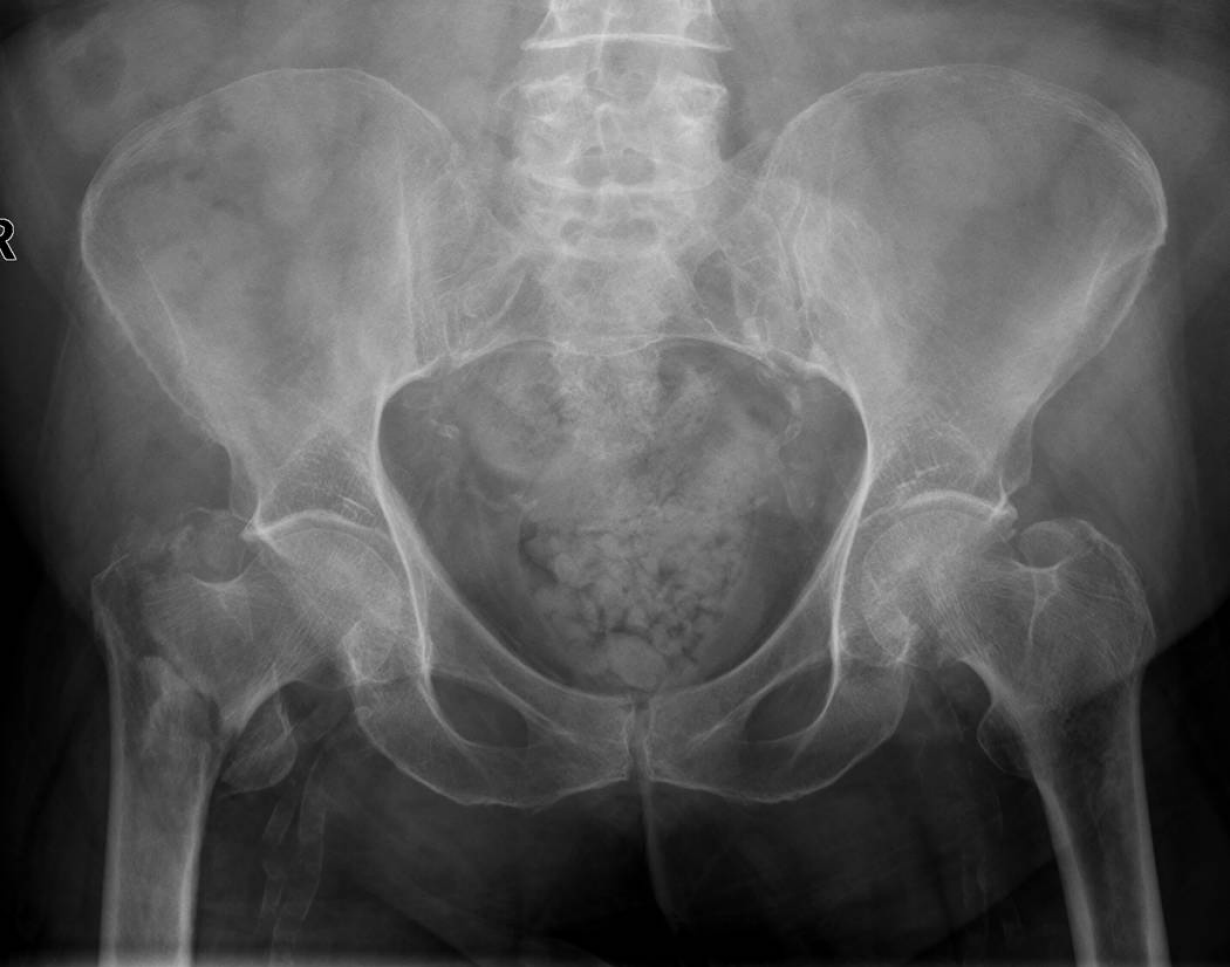
**Anterior-Posterior radiograph demonstrating a three part intertrochanteric hip fracture on the right side**. Discontinuety of the medial contex is seen with the less trochanter fracture fragment.

**Figure 2:**
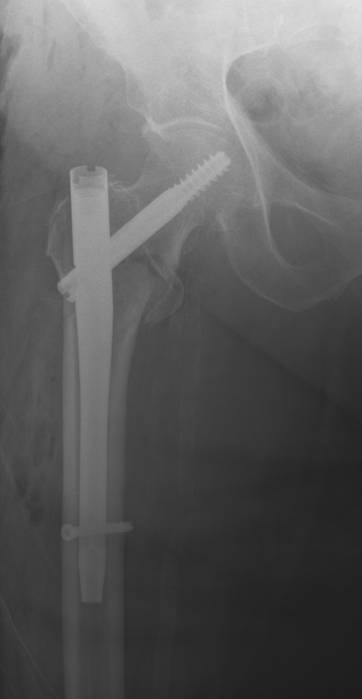
**Post-operative AP radiograph showing the reduction of the three part hip fracture with placement of the short Gamma 3 intrameduallary nail**.

**Figure 3:**
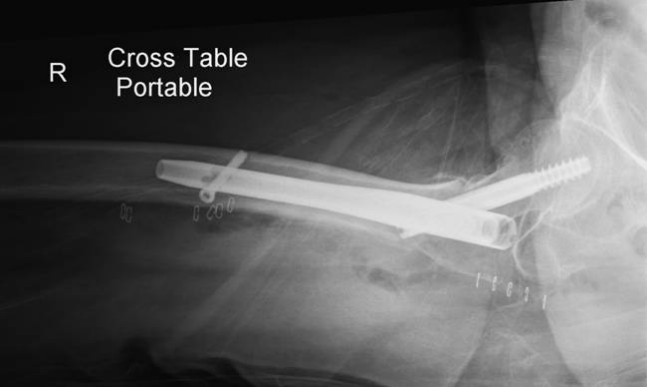
**Post-operative Lateral radiograph showing the reduction of the three part hip fracture with placement of the short Gamma 3 intrameduallary nail**.

The patient was a 77 year old Caucasian female who experienced a mechanical fall onto her right side and presented to our emergency department. On physical examination, the patient had a shortened and externally rotated right lower extremity that was painful to log-roll and axial load. Otherwise the patient was neurovascularly intact. The initial radiographs showed a 3- part inter-trochanteric hip fracture with the lesser trochanter as a separate fragment (Fig. 1). The patient was taken to the operating room after medical clearance for a closed reduction and insertion of a short Gamma 3 nail (125 degrees × 11 mm × 180 mm, Stryker, Kalamazoo, MI) according to standard technique. A 100 mm lag screw was inserted close to the subchondral bone but without penetrating the femoral head with a total Tip to Apex Distance (TAD) of 13 mm (AP = 5 mm and Lateral = 8 mm). After confirmation of firm fixation of the lag screw, a distal locking screw was inserted in the dynamized position of IM nail. A set screw was then placed to prevent rotation of the lag screw. There was no intra-operative complications and post operative anterior-posterior and lateral hip radiographs are seen in Figs. 2 and 3. Post-operatively, the patient was discharged to rehab facility on day 3.

**Figure 4:**
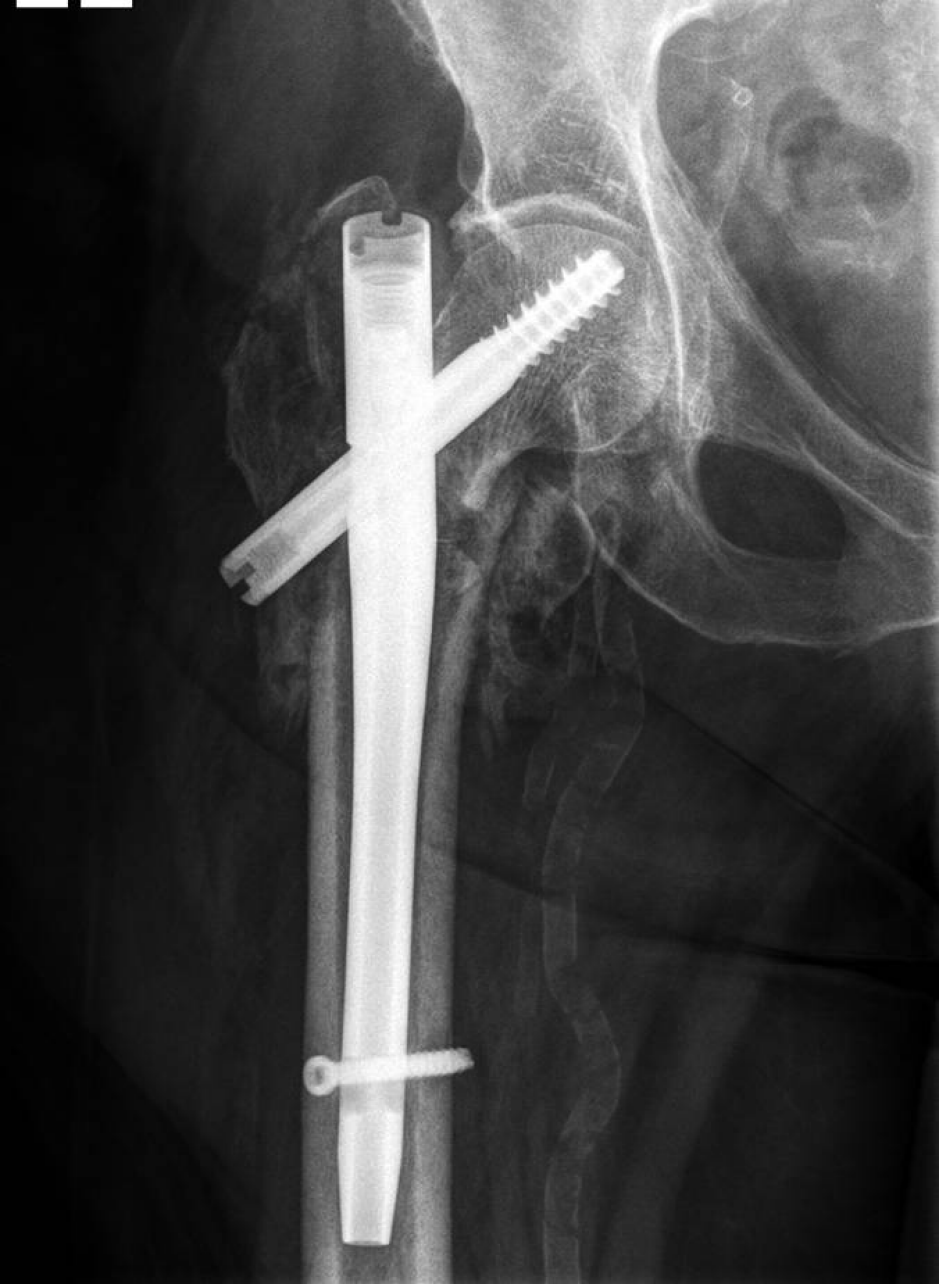
**Anterior-Posterior radiographs at the six week post operative clinic visit shows compression of the Lag screw without medial migration**.

**Figure 5:**
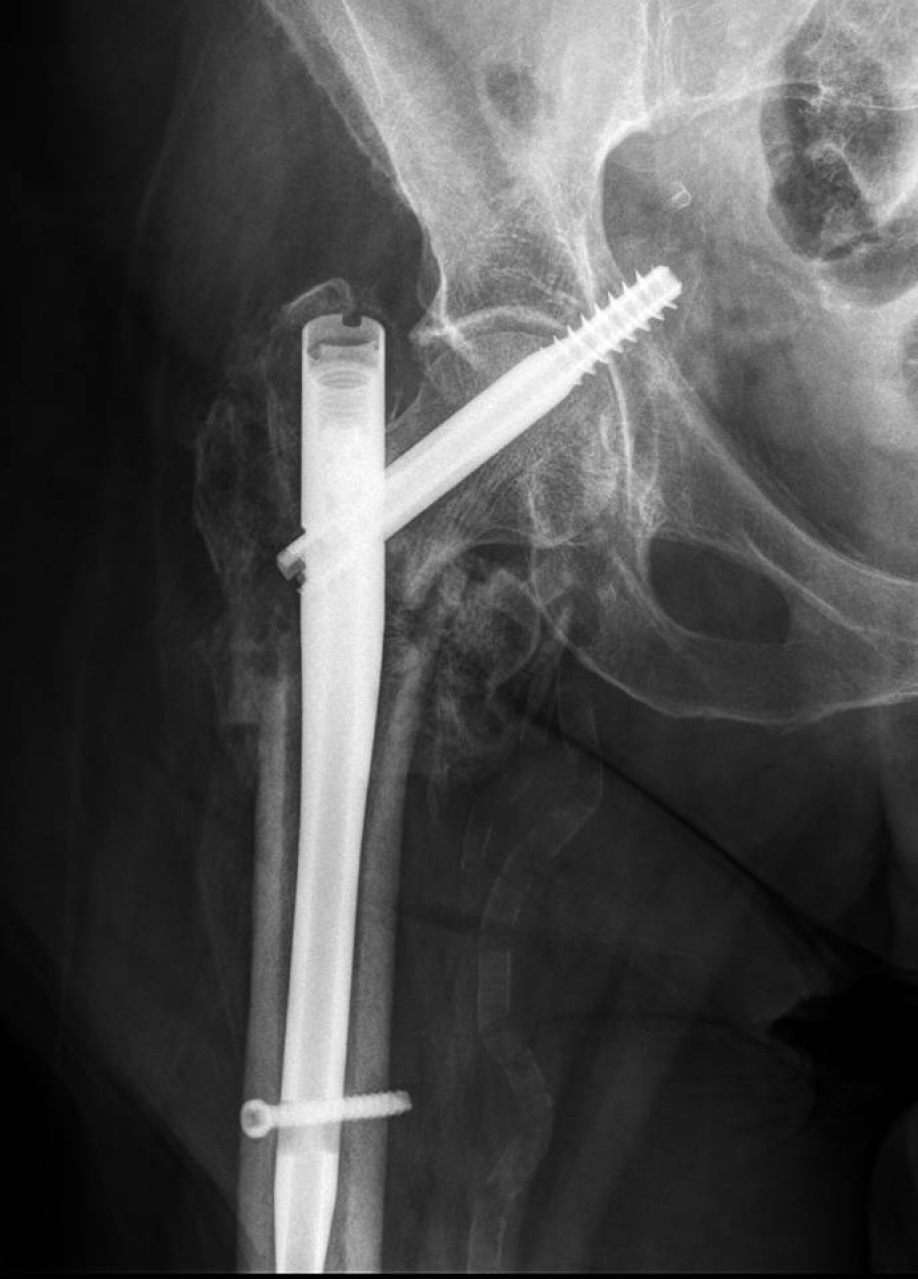
**Medial migration of the Lag Screw into the pelvis at the ten week post operative clinic visit**.

**Figure 6:**
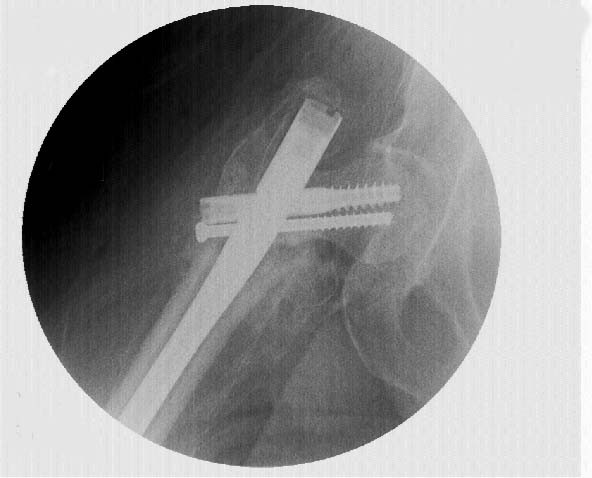
**Intra-operative fluoroscopic image at the time of revision surgery**. A shorter Lag Screw was placed and another accessory cannulated screw placed anterior and in parallel to the Lag Screw.

**Figure 7:**
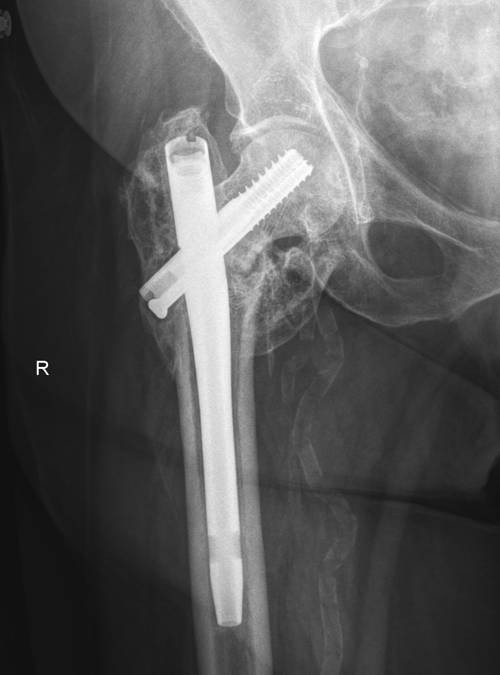
**Most recent AP hip radiograph at the one year post revision clinic visit shows good implant position and a well healed intertrochanteric hip fracture**.

On the sixth week post operative visit, the patient had no complaints and she has been weight bearing as tolerated. Anterior posterior right hip radiograph shows some callus formation and compression of the lag screw without medial migration (Fig. 4). At the ten week follow up visit, right hip radiographs revealed that the lag screw along with the short IM nail construct had migrated medially through her femoral head and through the medial wall of her acetabulum (Fig. 5), however, the patient have been ambulating on her right lower extremity and did not complaint of hip pain or have any neurovascular deficits. Patient was subsequently admitted and underwent revision surgery with removal of the lag screw and placement of a shorter lag screw after intra-operative stress confirmation of no visible fracture motion. An accessory cannulated screw was then placed anterior and parallel to the Lag Screw to provide de-rotational component (Fig. 6). The distal locking screw of the IM nail was then removed to provide axial dynamization of the nail. The patient tolerated the procedure well and continued to do well clinically at the one year follow-up (Fig. 7) and currently back to full activities with full weight bearing and cane assisted ambulation.

## Disscusion

Intertrochanteric hip fractures are very common among the elderly [13,14]. There are many different fixation devices developed for the management of these fractures, with majority of them belonging to either the intrameduallary or the sliding hip plate category. The advantages of intrameduallary fixation system are decreased intra-operative blood loss and operating room time with immediate load-bearing. Better clinical outcomes have also been reported when utilized in the unstable fracture pattern when compared to the sliding hip plates [4,15]. However, higher complication rates associated with distal femur fracture and lag screw cut out have also been reported with the IM system [4].

Several rare complications related to the lag screw in an IM hip fixation system have been seen in the literature as case reports. Tauber et al. reported a case of sigmoid perforation with free medial migration of the lag screw in a Gamma nail. The complication presented 8 weeks post operative without any history of trauma. The sigmoid perforation presented at 12 weeks as retroperitoneal abscess and sepsis. No intra-operative complications were reported from surgery and the authors could not explain the cause of this complication [8]. Another report in the literature described spontaneous extracorporeal extrusion of the Proximal Femoral Nail lag screw from the body of a patient over one year from the date of surgery. Given that the PFN system has no build in mechanism to stop lateral migration of the lag screw, the authors urged for a modification of the PFN system [11,16]. Another case of lateral lag screw migration without extrusion from the skin in the setting of a minor trauma has also been reported [10].

Walking and normal weight bearing subjects the implant and bone surface to combined axial and torsional load that may play a role in lag screw migration. Werner-Tutschku et al. described a complication of the PFN when the superior anti-rotation screw penetration into the femoral head or pelvis while the distal femoral neck element (lag screw) migrating laterally. They termed this the "Z-Effect" which was seen in 7.1% of their cases (7/70) associated with the PNF with two femoral neck elements. The authors attributed this complication to bad primary reposition in varus which causes collapse of the fracture and sliding of the inferior neck screw laterally while the superior anti-rotational screw migrated medially [6,12]. This "Z-Effect" or medial migration of the femoral neck element (FNE) was recently analyzed biomechanically. Several different IM hip fixation systems where tested and medial migration of the femoral neck element was reproduced reliably in each. The authors found that certain fracture patterns (deficient lateral buttress or an unstable medial calcar) that led to nail toggling within the femoral canal are more likely to cause medial migration of the FNE in implants with only one FNE. Preventing nail toggling was able to prevent medial migration of the FNE. However, the exception to the rule was seen in the PFN with two FNEs, as preventing nail toggling did not prevent medial migration of the distal Lag Screw. Thus the authors concluded a different mechanism other than nail toggling may be responsible for the "Z-Effect" seen in the dual screw PFN systems [6]. Another biomechanical study showed that specimens with the greatest mechanical mismatch between femoral head and neck exhibit lateral migration of the distal femoral neck element of a dual screw IM system. Specifically, migration is seen if the compressive strength of the femoral head is much greater than the femoral neck, which simulates fractures that have significant medial cortex comminution that are prone to varus collapse [17].

In the presenting case, the patient had an unstable intertrochanteric hip fracture with the discontinuity of the medial cortex. A short Gamma 3 nail was placed with the Lag Screw locked rotationally by the set screw. The distal locking screw was then placed in the dynamized position of the Gamma nail. We theorized that over time with the compression of the fracture and dynamization of the nail in the setting of an unstable fracture pattern, there was toggling of the nail within the intrameduallary canal which led to the medial migration of the Lag Screw with repeated axial loading. This is the mechanism as proposed by Weil et al. in their biomechanical study [6]. Further penetration of the femoral head and eventual migration into the pelvis are more likely to be seen with osteoporotic bone [17]. Intra-operatively during the revision surgery, the Lag Screw was felt to be well fixed into the acetabulum even in the setting of medial migration into the pelvis. The patient was asymptomatic, which lead us to believe that the Lag Screw may have migrated at the center of rotation into the acetabulum thus with hip range of motion, the patient did not experience significant pain. Furthermore, we stressed the fracture site intra-operatively after removal of the Lag Screw and there was no fracture motion, which indicated that the fracture was well healed. Therefore, decision was made to replace the longer Lag Screw with a shorter one. We also wanted a de-rotational component to the IM construct to further prevent spinning of the femoral head on the shorter Lag Screw, therefore, a second cannulated screw was placed into the femoral neck anterior and parallel to the shorter Lag Screw. Furthermore, the shorter Lag Screw was also locked rotationally by the set screw. The patient did very well with the revision surgery and was able to return to full activities.

## Conclusion

Although the apparent mechanisms of the femoral neck element (lag screw) medial pelvic migration are undefined, incidents occur in part due to poor device placement during surgery leading to varus collapse post operatively, unstable fracture patterns especially with comminution of the medial calcar or lateral cortex, and post-operative weight-bearing on osteoporotic bone have been reported as contributing factors in literature. The medial Lag Screw migration seen in our patient is likely a combination of the above mentioned factors. Even with application of the set cap which stabilizes the construct in torsion, axial load may still cause toggling of the nail within the femoral canal leading to medial migration similar to what is seen in the "Z-Effect". It's also important to demonstrate fracture healing by stressing the hip intra-operatively. If there is no fracture motion, then replacing the original Lag Screw with a shorter Lag Screw and using a cannulated screw as a de-rotational device can be used as a surgical management option. Attention should be paid to the position of the IM implant to minimize varus collapse in order to decrease risk of medial migration of the Lag Screw.

## Consent

Written informed consent was obtained from the patient for publication of this case report and any accompanying images.

## Competing interests

The authors declare that they have no competing interests.

## Authors' contributions

XL contributed to the data collection/interpretation, figures, and drafting/revising of the entire manuscript. MH and CK contributed to the figure collection and to the introduction and case presentation section. The patient was evaluated and operated by WL. The final manuscript was seen and approved by all authors before submission.
